# Study on the mechanical response of anticlastic cold bending insulating glass and its coupling effect with uniform load

**DOI:** 10.1371/journal.pone.0250463

**Published:** 2021-04-23

**Authors:** Xide Zhang, Jinzhi Liang, Dong Huang

**Affiliations:** 1 College of Civil Engineering and Architecture, Guangxi University, Nanning, China; 2 Key Laboratory of Disaster Prevention and Structure Safety of Chinese Ministry of Education, Nanning, China; 3 Ministry of Education Key Laboratory of Disaster Prevention and Structural Safety, Guangxi University, Nanning, China; University of Vigo, SPAIN

## Abstract

Cold bending is a characteristic of significance for the beautiful curved glass curtain walls, because it affects them in terms of energy-efficiency and cost-efficiency. The increasing engineering projects call for more special studies on the mechanical properties of cold-bent glass panels, especially when the walls are built by insulating glass that is currently widely used while its relevant research is very scarce. This paper is devoted to studying the mechanical properties of anticlastic cold-bent insulating glass while taking different factors into consideration, including glass thickness, cold-bent torsion rate and cavity thickness. 9 pieces of insulating glass were manufactured for anticlastic cold-bending test and their coupled effect with identical load is also studied, and numerical finite element analysis sessions were carried out to simulate the experimental results for each one of them. Further, we analyzed the stress distribution performance of the sample pieces under cold bending and a uniform load, followed by discussions about stress transfer controls in glass plates. The results showed that the cold-bent control stress is on the surface with direct loads from cold bending and close to the cold-bent corner on the short edge, and it is transferred from the parts around the corner to the center when the uniform load plays a leading role in generating stress. This transfer could occur under a relatively small load with a small cold-bent torsion rate. A higher cold-bent torsion rate in cold bending contributed mostly to greater center stress in the glass, and as the glass thickness grows, stress and deflection at the plate center would significantly drop. However, the effect of cavity thickness on the anticlastic mechanical response of insulating glass was found to be trivial.

## 1. Introduction

In recent years, the applications of curved glass curtain walls are growing wider and wider. Traditionally, curved glass is made by plane fitting or heat bending. The first approach would produce a rough curved surface by plane splicing, achieving poor outcomes with a large curvature. The second method is one of the most used to manufacture small objects with a large curvature [1], but it requires a large number of molds to bend glass plates, and the overall complex procedure needs to be carried out at a high temperature, usually above 550°C[2]. The shortcomings of heat bending often make people unsatisfied. The main reason is that a slight unevenness of the glass during the heat bending process may affect the optical quality [3]. For plates with different curvatures, different molds are required, so this method is neither energy-saving nor economical [4], and the transportation conditions and delivery time are also unsatisfactory [3–5]. However, a rather new technique named cold bending can effectively evade the shortcomings of the other two methods mentioned, where the glass can be bent under ambient temperature on the construction site [5]. In addition, prefabrication of glass product batches can be performed in the factory [6]. This technique takes advantage of the ability to bend and elasticity of flat glass [3], and after reasonable clamping or applying external force, a flat glass piece shall be forced to bend into the geometric shape required. By this technique, the number of problems about bad optical quality, probably as a result of permanent deformation caused by heat bending, is significantly reduced [7], which is due to the cold-bent glass is always reversibly deformed as demonstrated by curvatures within the elastic range. Furthermore, cold bending is advantageous in terms of low cost [9], low energy consumption, environment friendliness, and short time of production [8]. It has been applied in many engineering projects, such as Strasbourg Railway Station in Alsace, France [9]; Victoria & Albert Museum in London, UK [7]; Christoph Dengler Seele glass bridge in Germany [10], etc.

Since the elasticity of the glass is critical in cold-bending, there’s no doubt that permanent stress will be generated inside the glass unit [1]. Previous researches on cold-bent glass focused on the stress and deformation of the glass plate, including the buckling deformation. For cold-bent monolithic glass, the theory of plates and shells is applicable, and the alterations in boundary conditions and cold bending load are significantly influential on the plate stress and deformation. Compared to its monolithic counterpart, cold-bent laminated glass possesses better performance and lower costs [11], leading to its wider application in cold bending. On the other hand, the interlayer material within laminated glass is a hyperelastic or viscoelastic polymer, which deforms along and interacts with the glass during cold bending, resulting in, difficult theoretical analysis and prediction of the overall stress. As for structures made by insulating glass, spacer bars, including silicone sealants, aluminum spacer bars, butyl tapes, etc., are locally connected to the surrounding edges, where the stress and deformation become more complicated in cold bending. In addition, it is rather impossible to use one single method to study the cold-bent insulating laminated glass, and there are even more unpredictable difficulties on the way when studying its mechanical response.

Current researches are relatively limited for the technique of cold bending. What’s more, even in those studies focusing on such technology, the objects of study are mostly monolithic and laminated glass, such as the study on the buckling phenomenon of monolithic glass by double curved cold bending [12], and the analysis of shear stress of laminated glass [13, 14]. Although theoretical analysis and numerical simulation occupy a large and important part in research on cold-bent glass, there is a trend in recent years that cold-bent glass can be studied through experiments, such as Spagnoli A et al. [13] studied the geometric nonlinear bending of plates, and Quaglini V et al. [1] studied the mechanical response of cold-bent parabolic-shaped glass panels, while the relevant research is very scarce for insulating glass, especially in the use of experimental methods. However, insulating glass is largely used in curtain walls and building envelopes in most countries and regions [14], as it can greatly reduce the energy consumption of the whole building while providing excellent thermal insulation compared to the other two. Moreover, in some other countries, relevant national and industry standards require insulating glass to be mandatory in cold areas(such as China [15]). Most recent researches on insulating glass focus on heat and sound insulation, as well as durability [16, 17] while not paying adequate attention on cold bending, especially the commonly used anticlastic cold bending. Anticlastic cold bending is one of the most common cold bending curved shapes and its specific form can be found in ref. [15]. A typical anticlastic cold bending is that one corner of a rectangular plate produces cold bending displacement, and the other corners are fixed. In fact, anticlastic glass plays an important role in high-tech, complex, and beautiful curved glass curtain walls.

When applying cold bending to insulating glass, plate stress is closely related to spacer bars and greatly affected by the bending pattern. The deformations of each component in insulating glass are different under a large curvature. If excessive dislocation between two glass sheets appears, the risk of damage for spacer bars exists, even resulting in overall performance failure [15]. Besides, cold-bent stress in the glass is present in all working environments for a long time. Under external load, e.g., the wind, the stress will superimpose and couple, leading to a change in the bearing capacity.

Therefore, we can see that it is particularly important to study the mechanical response of cold-bent insulating glass. This paper looks into the mechanical response of frame-supported insulating glass made by anticlastic cold bending under superimposed uniform loads through experiments and finite element simulation with a focus on stress distribution and the impact of different relevant factors, such as glass plate thickness, cold-bent torsion rate, and cavity thickness, on stress and deflection.

## 2. Experiments

### 2.1 Sample design

There were three factors and three levels in the experiment, so the orthogonal table *L*_9_(3^4^) was used in the design of the samples basing on the principle of the orthogonal experiment, and 9 glass samples were made for cold bending test, and relevant parameters were shown in Table 1. The insulating glass sheet in the test was provided by the glass manufacturer and the type was tempered glass. The composition was mainly Na_2_SiO_3_, CaSiO_3_, SiO_2_, or Na_2_O·CaO·6SiO_2_. Since the glass material has not changed before and after tempering, except for the inconsistency in strength, the typical physical-mechanical parameters are no different from ordinary glass. The Chinese standard [19] believes that tempered glass has the same elastic modulus and Poisson’s ratio: Young’s modulus *E* = 72GPa; Poisson ratio *υ* = 0.22. The size of each insulating glass piece was 2200mm×1200mm. The cold-bent torsion rate *β*, the glass plate thickness *t* and the cavity thickness *t*_*c*_ were selected as test variables after careful consideration of the internal stress in the samples. There is no precise description of the index of anticlastic cold bending. To describe the degree of cold bending of insulating glass, we refer to the warpage measurement of PCB board in the IPC-TM-650 test methods manual and define the cold-bent torsion rate *β* as:

β=s/2c×100% (1)

where *s* is the maximum cold bending displacement of the free corner point; *c* is the diagonal length of the glass piece.

**Table 1:** Test parameters.

Specimen	Size[mm]	*t*[mm]	*t*_c_[mm]	*β*[%]	*s*[mm]
SJ1	2200×1200	4	9	0.0	0
SJ2	2200×1200	4	12	0.3	15
SJ3	2200×1200	4	15	0.6	30
SJ4	2200×1200	6	9	0.3	15
SJ5	2200×1200	6	12	0.6	30
SJ6	2200×1200	6	15	0.0	0
SJ7	2200×1200	8	9	0.6	30
SJ8	2200×1200	8	12	0.0	0
SJ9	2200×1200	8	15	0.3	15

In the experiment, we chose *β* to be 0%, 0.3% and 0.6%, respectively. From Eq (1), *β* was calculated to be 0.3% and 0.6%, corresponding to the maximum displacements of the free corner *s* that were 15mm and 30mm, respectively. Additionally, we selected the glass thickness to be either 4mm, 6mm or 8mm, and the cavity thickness was determined to be either 9mm, 12mm or 15mm according to Chinese code [18]. All the thickness numbers are the most common in practice.

**Fig 1:**
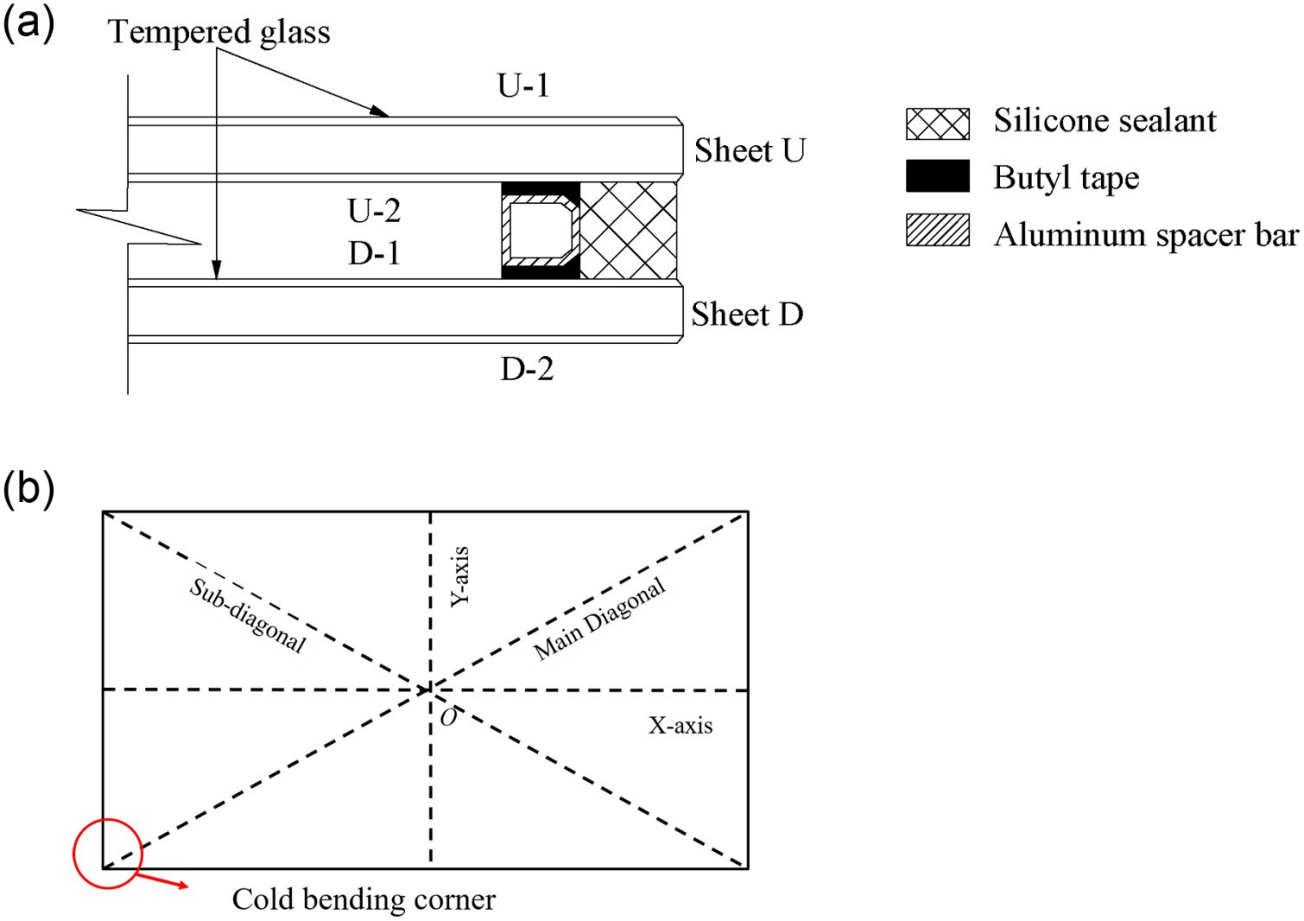
The nominations of the insulating glass. (a) The nominations of glass surfaces; (b) The nominations of axes and diagonals.

The nominations of the insulating glass samples were shown in Fig 1: (1) the upper and lower glass sheets were indicated by the letters “U” and “D”, respectively; (2) the numbers "1" and "2" were used to represent the upper and lower surfaces of the glass, respectively. For example, "U-1" stands for the upper surface of the upper glass sheet; (3) the center point of the glass surface was defined as the origin *O*, the axis along the long side was defined as the *x*-axis, and the axis on the short side was defined as the *y*-axis. In addition, the diagonal line on the cold-bent corner was noted as the Main Diagonal, and the other one was named the Sub-diagonal.

### 2.2 Experimental settings

**Fig 2:**
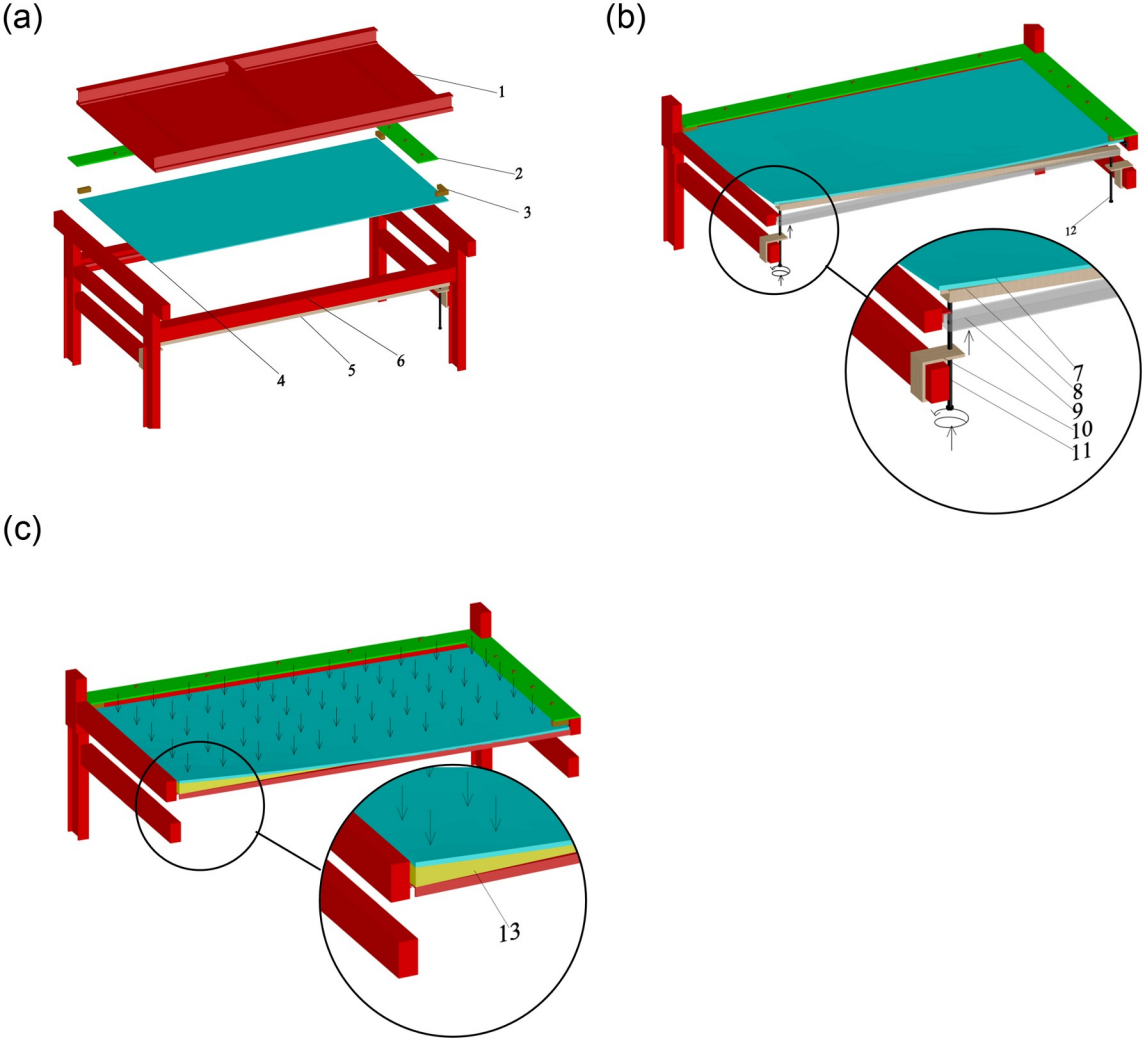
Test device. (a) The assembly of the cold bending device. (b) Schematic diagram of the cold bending process. (c) Schematic diagram of the uniform load test. The symbols in figures stand for: 1. Reaction frame, 2. Fixed steel plate, 3. Timber block, 4. Glass, 5. Cold bending loading device, 6. Support frame, 7. Glass, 8. Square steel pipe (final position), 9. Square steel pipe (initial position), 10. Fixed buckle, 11. Long screw (cold bending end), 12. Long screw (fixed end), 13. Wedge-shaped inclined brace.

**Fig 3:**
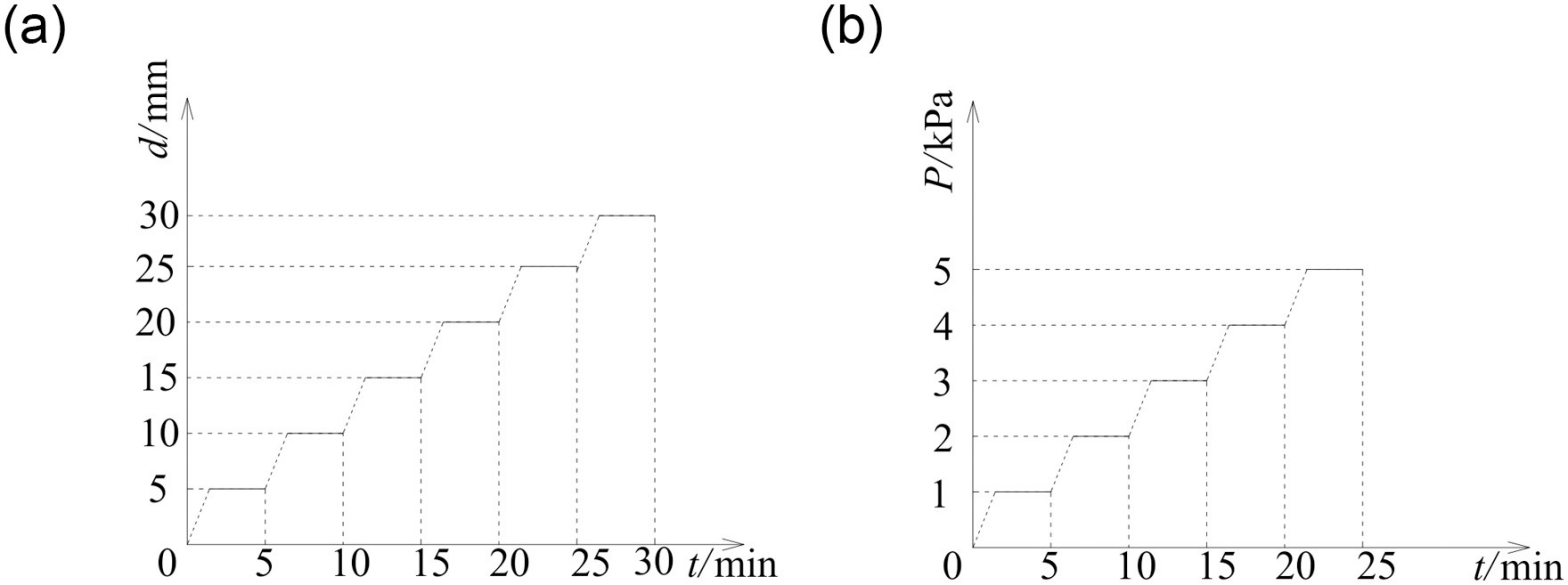
Loading history curves. (a) Cold bending. (b) Uniform load.

A special test device was designed and built to simulate the manufacture and working status of cold bending glass curtain walls in real-world projects. It is capable of performing cold bending tests and uniform load tests for frame-supported glass panels. Fig 2(a) was the assembly diagram of the test device, which consisted of a load reaction frame, a steel plate fixture and a support frame for glass plate and cold bending loading device. Among them, the cold bending loading device was specifically designed for glass plates, which was composed of a square steel pipe, long screws and fixed buckles. Two ends of the square steel pipe were connected with a long screw by two fixed buckles, which were located on the support frame as shown in Fig 2(b). Furthermore, the load reaction frame could be removed and cut along the free long edge of the sample plate for better observation. Installed under the long edge of the sample plate, one end of the load reaction frame was placed below the cold bending corner, while the other was located beneath the fixed corner. Both ends were fixed to the short beam at the bottom of the support frame with fixed buckles that connect the long screw and the steel pipe, so that the latter can move up and down freely and be lifted into contact with the glass after the sample was fixed on the supporting frame. Then the square steel pipe at the fixed corner end was kept still, while the other end can be lifted by rotating the screw (The screw 11 in Fig 3), pushing the glass plate upward for cold bending. The square steel pipe was kept in contact with the bottom of the sample glass plate along the cold bending procedure. Consequently, rather than directly applying force on the cold bending corner on the plate, square steel pipes can prevent stress concentration or even damage due to local forces. Such device has been used in real-world engineering.

**Fig 4:**
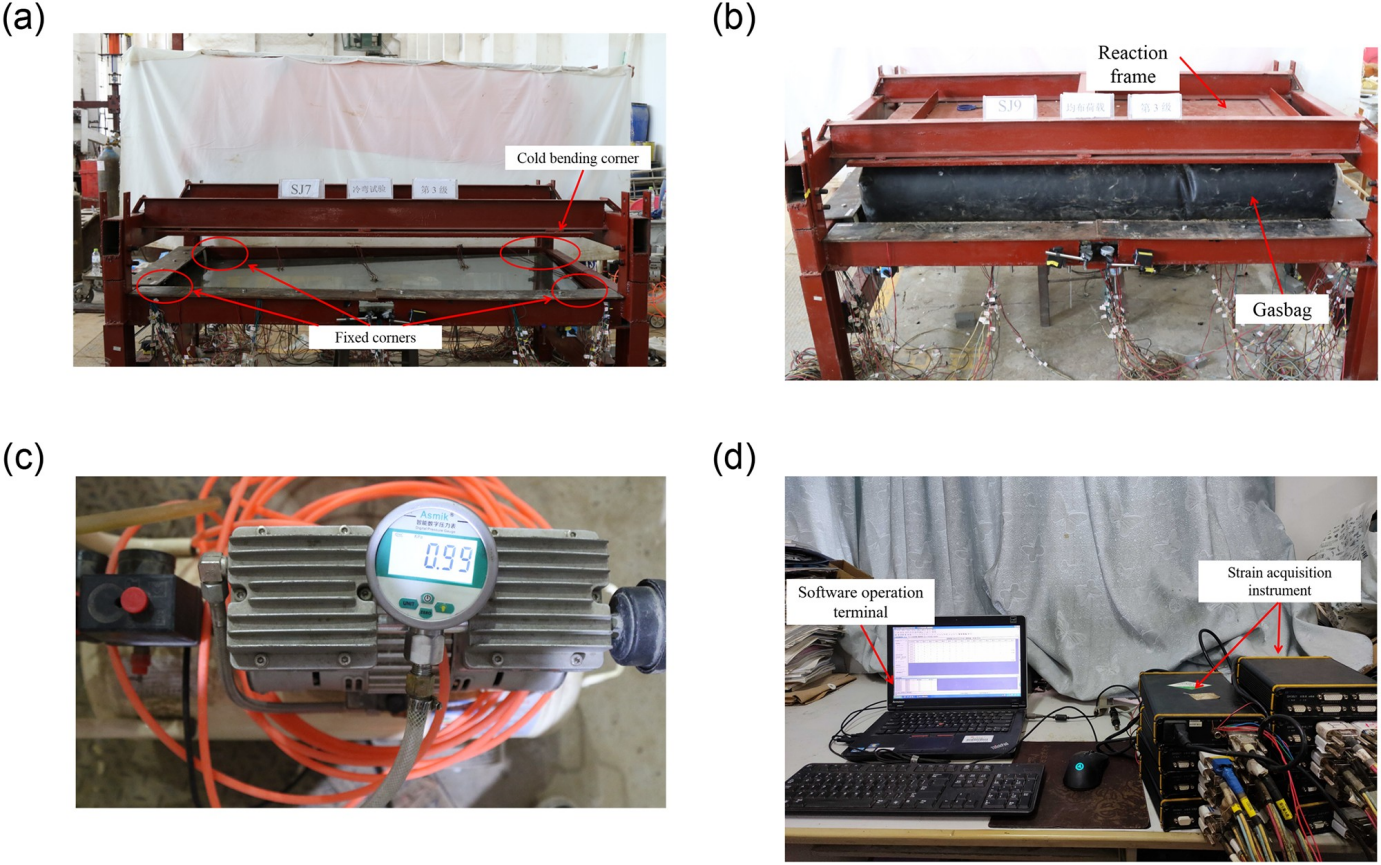
Real shot diagram and data acquisition system during the test. (a)Loading of cold bending; (b) Simulation of uniform load with gasbag; (c) gas pump and pressure gauge; (d) Strain acquisition system.

Two tests were performed in the experiments, namely the cold bending test and the uniform load test. The construction of the cold bending test was shown in Figs 2 and 4(a), and view of Fig 4(a) was that the cold bending corner of the device in Fig 2(a) and 2(b) had horizontally rotated to the upper right corner. A pair of adjacent glass edges were placed on the support frame, the latter of which were fixed by clamping timber blocks along with steel plate fixtures. The other pair of adjacent edges were free, the intersection of which was the cold bending corner. In addition, rubber pads were put between the glass and the supporting frame (or timber blocks) to avoid local stress caused by direct contact. When the glass plate was successfully fixed on the cold bending device, the steel pipe was pushed up by screwing the long screw to achieve a pre-determined cold bending displacement, which is categorized into several levels. The difference between two adjacent levels of displacement was 5mm, which can be monitored by a sensor at the cold bending corner. The cold bending test was conducted to study the internal stress distribution of the sample insulating glass sheets during cold bending. The loading history curve was shown in Fig 3(a). Regarding the loading rate, the study [2, 4] demonstrated that the loading rate of the cold bending glass panel will not affect the geometric response, so a low uniformly loading rate was used to load the cold bending displacement, and the same as the subsequent uniform load test.

The uniform load test was performed to simulate the coupling effect of cold bending and uniform load, as shown in Figs 2(c) and 4(b). The view of Fig 4(b) was the same as that of Fig 4(a), but no cold bending loading device, including screws, was installed, and a gasbag was applied as a uniform load. The maximum stresses and deformations of insulating glass in use mainly come from environmental/climatic loads of external pressure, such as wind load [17]. Therefore, it is necessary to study the influence of wind load on the stress and deformation of cold-bent insulating glass. After the cold bending test came to an end, a wedge-shaped inclined brace corresponding to the cold bending displacement was placed between the sample and the support frame to maintain the cold-bent shape. Actually, the wind load had different directions on the glass and the load perpendicular to the glass surface was the most unfavorable, so the effect of the load perpendicular to the glass surface was studied in the experiment and simplified by a uniform load with an inflatable gasbag. It means that the gasbag was pressurized by the gas pump, and the pressure value was monitored by the pressure gauge to simulate the effect of uniform wind load. The gasbag was installed between the sample plate and the reaction frame to represent a uniform wind load on the concave surface, as the most mechanically unfavorable condition. A Chinese industry standard [19] on the performance of wind pressure resistance showed that when such performance of exterior windows reaches the highest level 9, the corresponding wind load is 5kPa, which we used as the maximum test load, and the loading history curve was shown in Fig 3(b).

Three rectangular strain gauges of BX120-3CA (3X2) were used in the test, produced by Tai Zhou Huangyan Juxing Testing Instrument Factory in Zhejiang Province, China. The resistance was 120Ω and the sensitivity coefficient was 2.08±1%. The displacement gauges were digital indicators with the specification of (0–50) mm. As for the calibration value of the strain gauge and displacement gauge, the measurement uncertainty of the stress gauge and displacement gauge was 0.5% and 6.4μm, respectively.

**Fig 5:**
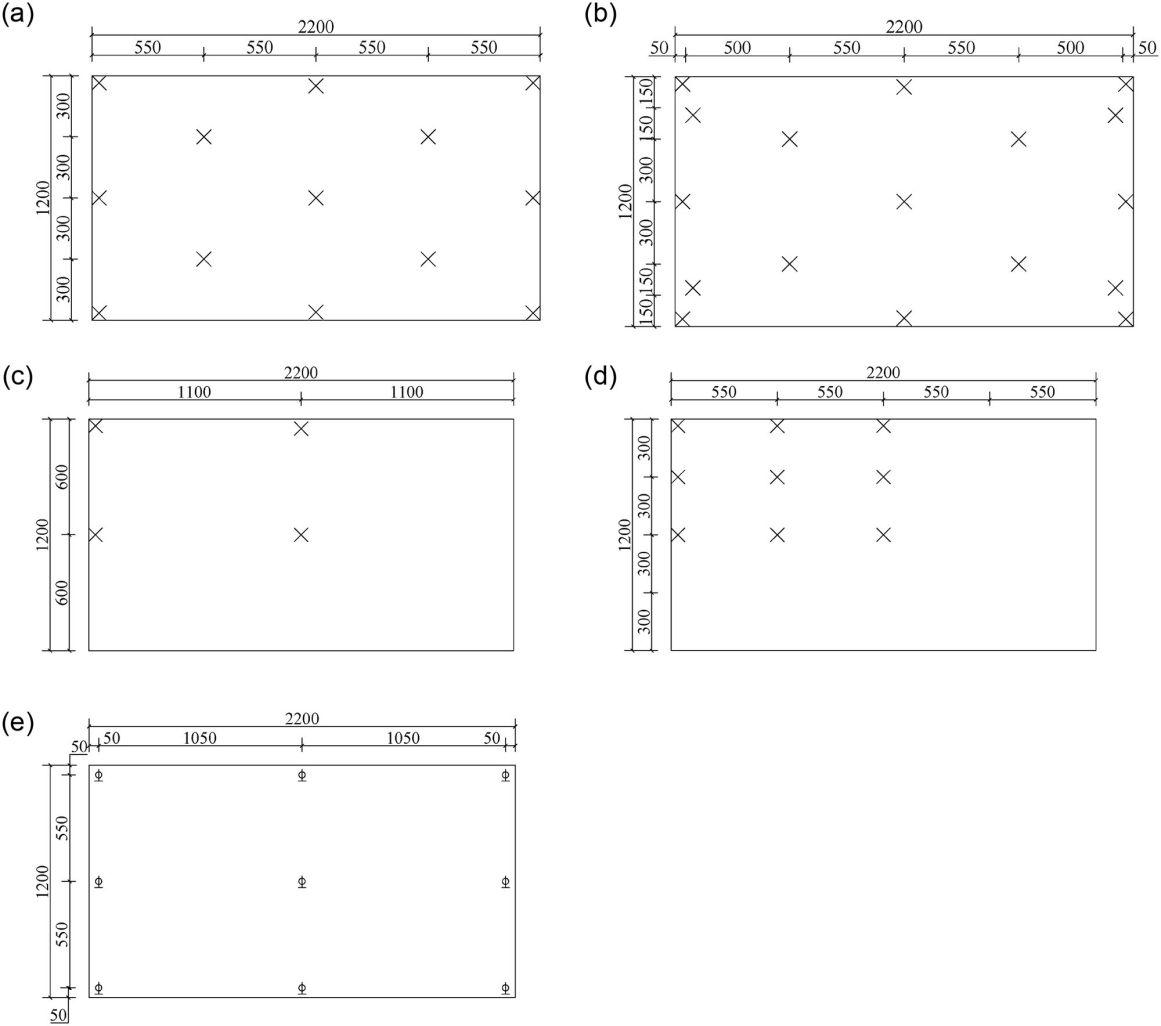
Layouts of measuring nodes. (a) Strain measuring nodes for cold-bent samples on surfaces of U-1 and D-1; (b) strain measuring nodes for cold-bent samples on surfaces of U-2 and D-2; (c) strain measuring nodes for non-cold-bent samples on surfaces of U-1 and D-1; (d) strain measuring nodes for non-cold-bent samples on surfaces of U-2 and D-2; (e) displacement measuring nodes.

The detailed layout of measuring nodes of stress and displacements was given in Fig 5. There were 19 nodes to measure stress on U-1 and D-1 surfaces, respectively, which were located at the center, four corners and the midpoints of each edge. The strain data were collected by the strain testing system. In addition, 13 stress measuring nodes were marked on the surfaces of U-2 and D-2, respectively. Three non-cold-bent samples, namely SJ1, SJ6 and SJ8, were used for comparison with cold-bent ones and nodes for stress measurement were placed symmetrically on the 1/4 glass plate. Moreover, 9 displacement sensors were evenly installed on the surface of the sample to obtain the deflection data, and they were set at the four corners, the midpoint of each edge and the plate center.

The test strain data was collected by the DH3821 static strain test system produced by Jiangsu Donghua Testing Technology Co., Ltd. (in China), with a sampling frequency of 2 Hz. It consists of two parts, namely the software operation terminal, and the strain acquisition instrument which was composed of strain acquisition cases, strain gauges and connecting wires. The data could be collected by the strain acquisition instrument and then was transmitted to the computer for data processing through the software operation terminal, as shown in Fig 4(d).

## 3. Results and discussion

### 3.1. Results of cold bending test

**Fig 6:**
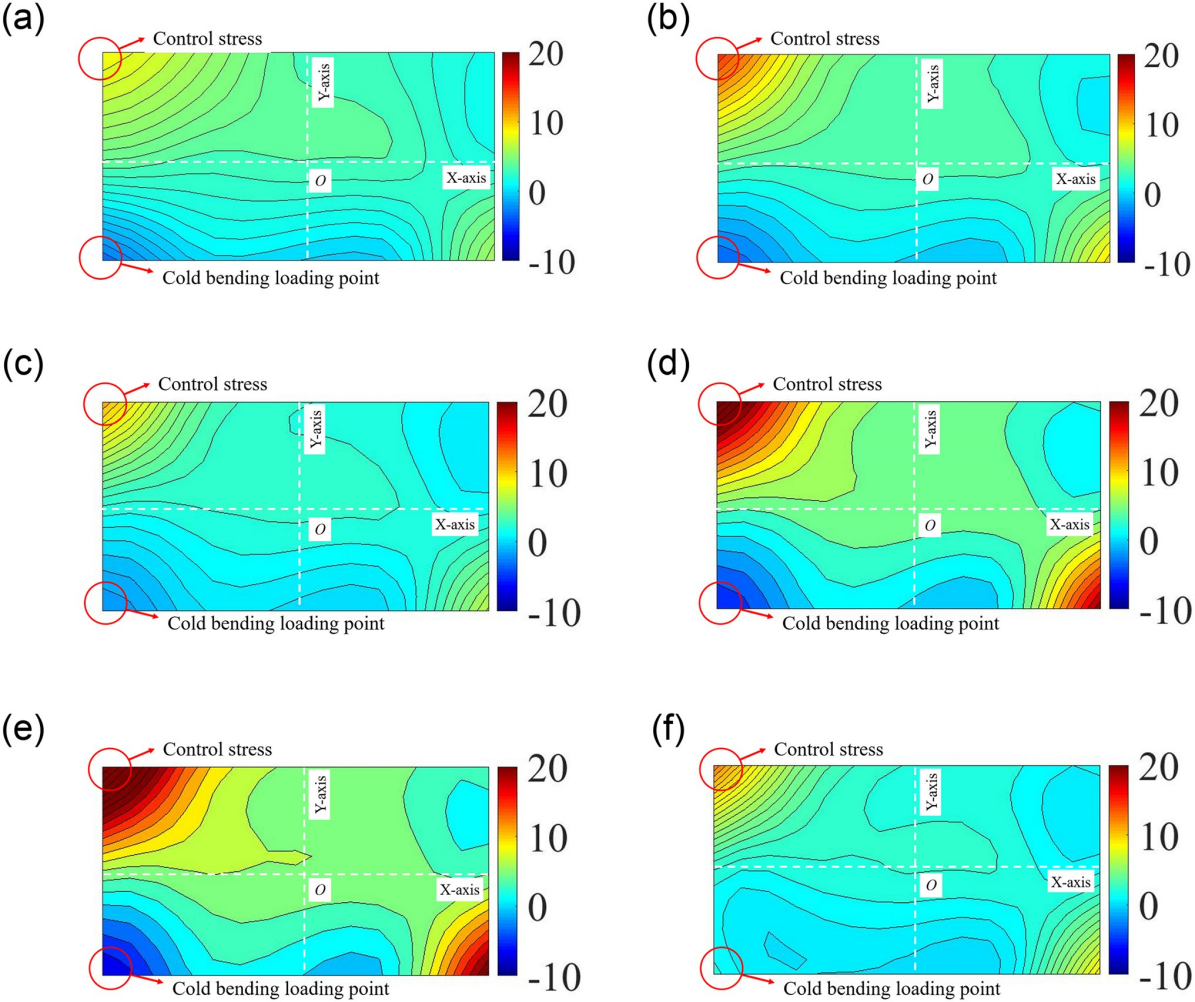
Cold bending stress nephograms on surface D-2 [MPa]. (a) SJ2; (b) SJ3; (c) SJ4; (d) SJ5; (e) SJ7; (f) SJ9.

The principal stress *σ*_1_ of each measuring node could be obtained by the strain test system to analyze the stress distribution on different surfaces. The maximum stress *σ*_1,max_ was found at the same position for all samples, which was near to the cold bending corner on the short edge of the surface D-2 and subsequently named the cold-bent control point. The *σ*_1,max_ values of each sample were shown in Table 2. The surface D-2 was directly subject to the vertical cold bending load, which explained its largest tensile deformation. Since the surface U-2 received the indirect transfer of the cold bending displacement and part of the strain energy on U-2 was absorbed by the spacer bars, its deformation and stress were not as strong as that of D-2. Another reason behind such findings is that the insulating glass exhibited interlaminar shear displacement. The stress difference between the two surfaces was 2%-18%, and the stress distribution on D-2 was shown in Fig 6. It could be seen that after cold bending, a rather uneven stress distribution was seen, where the stress on the ends of the Main Diagonal was lower than those on the Sub-diagonal.

**Table 2:** Maximum cold-bent stress of each sample.

Specimen	*t*[mm]	*t*_*c*_[mm]	*β* [%]	*σ*_*1*,*max*_[MPa]
SJ2	4	12	0.3	8.57
SJ3	4	15	0.6	15.68
SJ4	6	9	0.3	11.35
SJ5	6	12	0.6	26.83
SJ7	8	9	0.6	34.52
SJ9	8	15	0.3	12.91

The large cold bending displacement at the sample corner would cause geometric nonlinearity, resulting in a glass shape similar to a hyperbolic paraboloid where the Main Diagonal was squeezed and warped up while the Sub-diagonal was concave, a trivial equilibrium configuration characteristic of anticlastic cold bending curvatures. A previous study [13] had shown that when imposed displacement over the critical threshold, the Main Diagonal would become more concave, while the Sub-diagonal would become more rigid and tend to appear straight [14]. This phenomenon can be explained by the bifurcation instability in an equilibrium configuration. From the experimental results, we could deduce that the cold bending displacement applied here did not reach the critical threshold, which was demonstrated by stable glass samples.

### 3.2. Results of uniform load test

#### 3.2.1 Transfer of control stress

**Fig 7:**
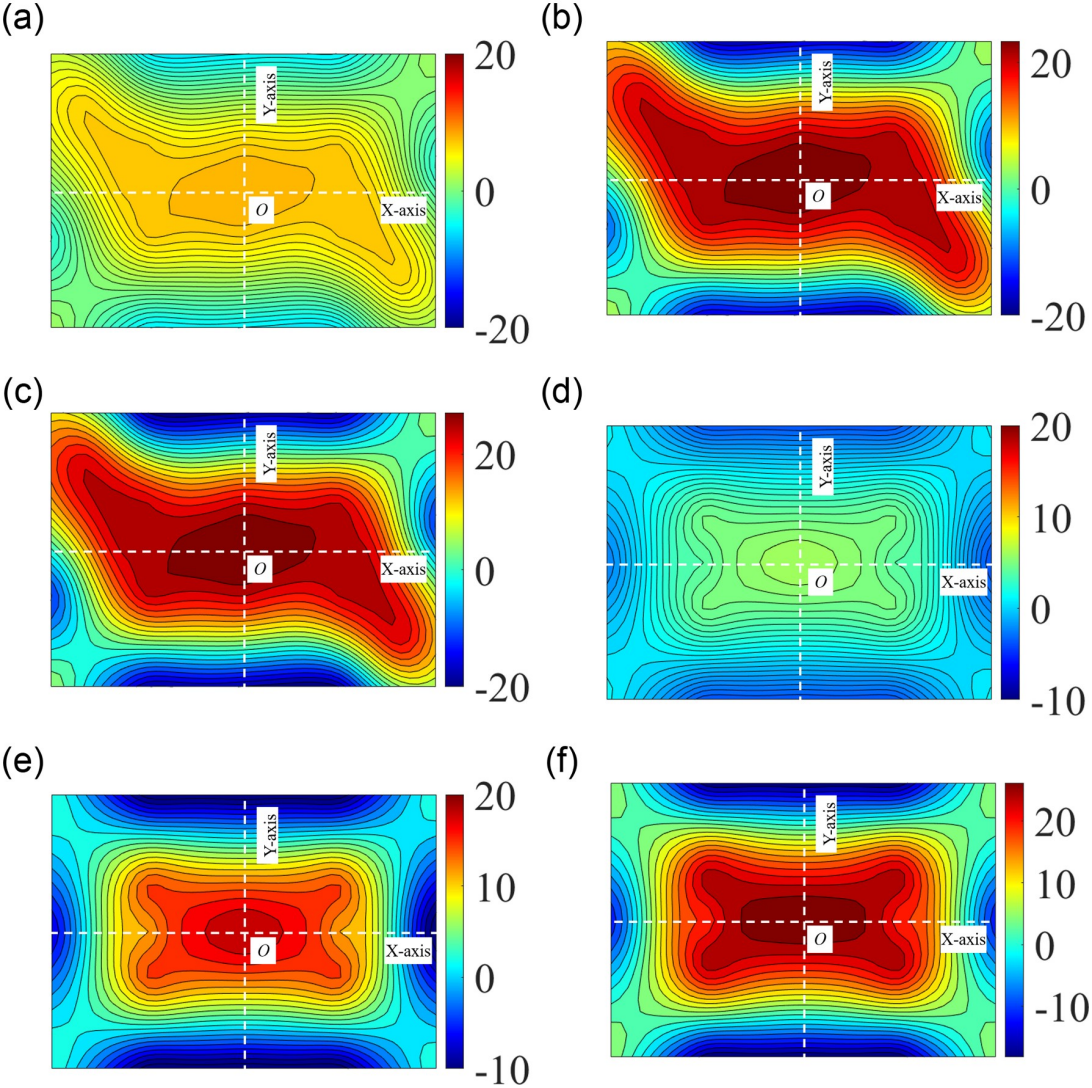
Stress nephograms in uniform load test on surface U-2 [MPa]. (a) SJ2,1kPa; (b) SJ2,3kPa; (c) SJ2,5kPa; (d) SJ1,1kPa; (e) SJ1,3kPa; (f) SJ1,5kPa.

**Fig 8:**
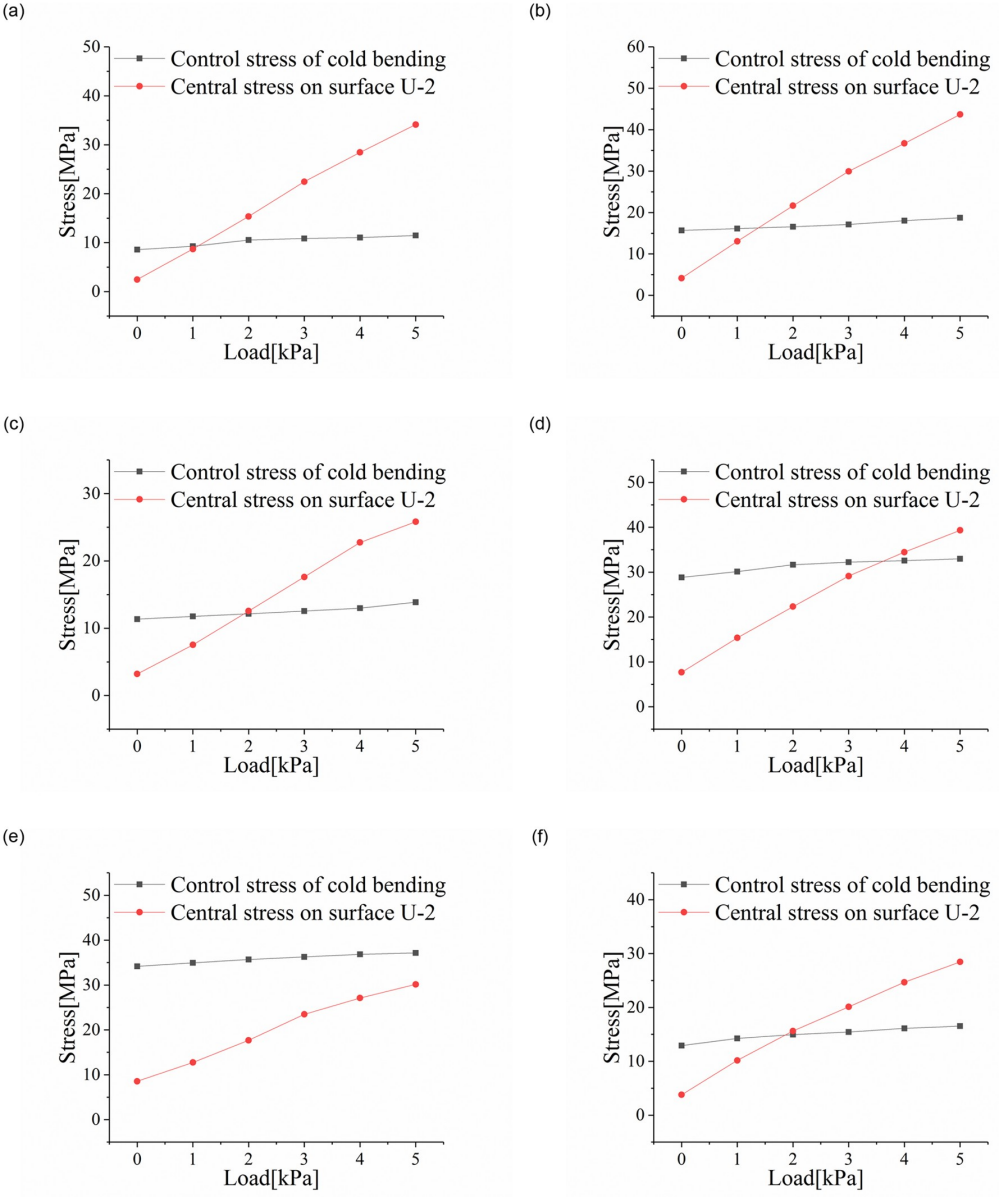
Stress on the Sub-diagonal of load test. (a) SJ2; (b) SJ3; (c) SJ4; (d) SJ5; (e) SJ7; (f) SJ9.

In this experiment, we used SJ2 as the typical example to analyze the stress distribution during the uniform load test after cold bending. The graph describing stress distribution changes along the test was shown in Fig 7(a)–7(c). The control stress distribution started on the surface U-2, which gradually became more and more uneven with the stress on the Sub-diagonal grew more and more prominent. At the same time, the control stress of most samples (except for SJ7) was transferred from the diagonal ends to the center area (Fig 8), which was directly related to the stress caused by a uniform load.

The stress distribution in non-cold-bent glass samples after applying a uniform load revealed a similar pattern, such as SJ1, where the center area appeared to contain the highest stress and was undergoing tension, while the edges were subject to small stress and were in a state of compression, as shown in Fig 7(d)–7(f). For the cold-bent samples, the stress in the center increased because of the superposition and coupling effects of the cold bending and uniform load. Moreover, if a uniform load had a greater impact on the stress than cold bending, the cold-bent control stress at the ends of the Sub-diagonal would be smaller than that in the central area. That is to say, judging from the experimental results, a uniform load was confirmed to play a leading role in the sample stress, leading to the control stress transferring to the central area and a tension zone of the central area extended to both ends.

**Fig 9:**
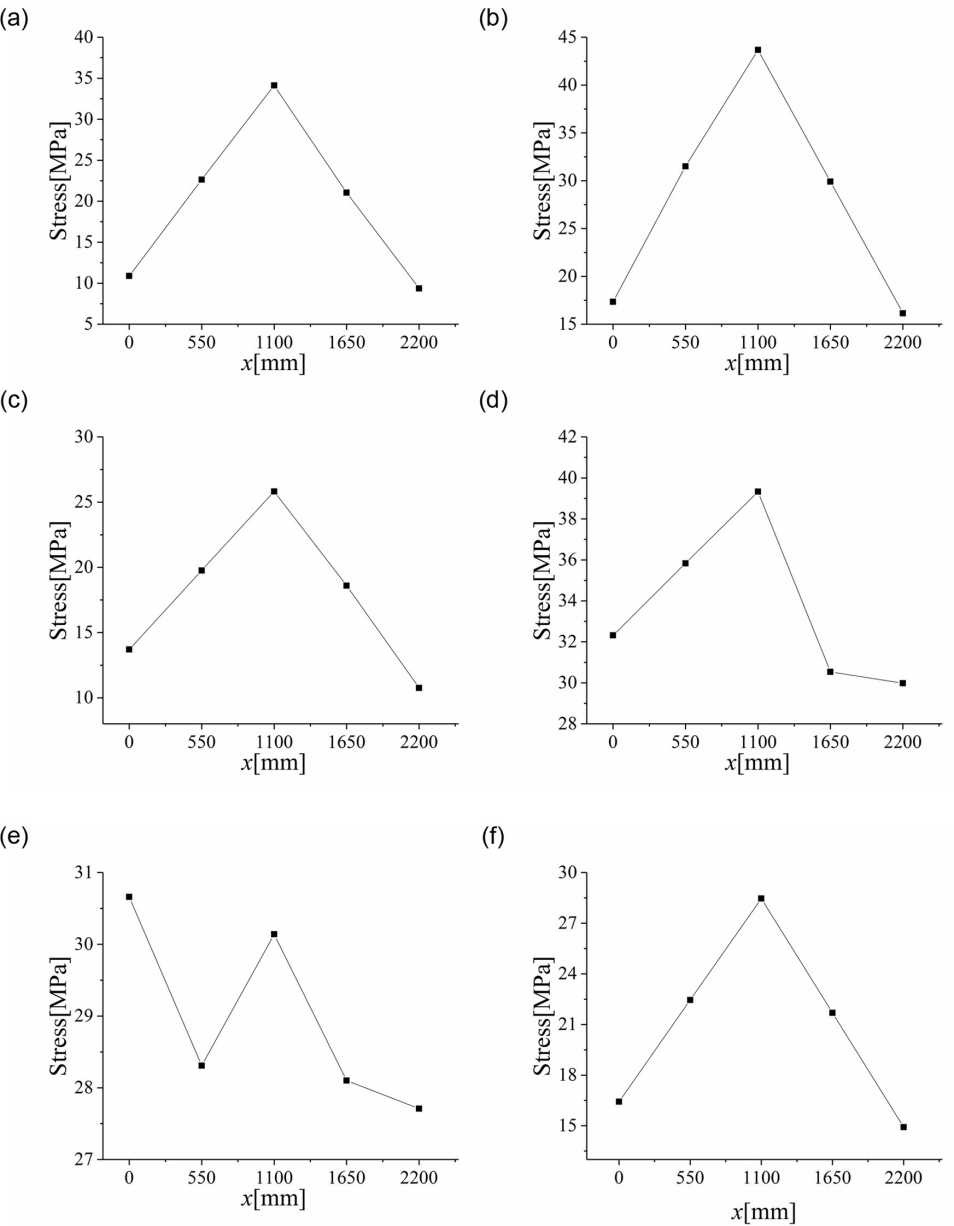
Correlation curves of control and central stress values with a uniform load. (a) SJ2; (b) SJ3; (c) SJ4; (d) SJ5; (e) SJ7; (f) SJ9.

Changes in extreme stress levels at cold-bent control points and the plate center were shown in Fig 9, where the control stress transfer of the insulating glass samples could be obviously observed. During the entire loading experiment, the stress at the cold-bent control points did not change much, but center stress rose rapidly and gradually took a prominent role (except for SJ7). In other words, for insulating glass, the control stress was transferred to the plate center with a relatively small cold-bent torsion rate after the implementation of a small load (less than 2kPa).

Experimental results showed that the transfer of control stress occurred when the uniform load played a leading role in causing stress on the glass plate. When the uniform load was not dominant to initiate stress, the stress at the control point did not transfer, but it still showed a tendency to expand to the central area in the tension zone, especially with a large cold-bent torsion rate and a thick glass plate. Evidence for this opinion is the results obtained from SJ7, whose maximum principal stress point was still at the cold-bent control point.

#### 3.2.2 Extreme stress and deflection at plate center

The maximum control stress of cold-bent insulating glass was found on the surface U-2, which in turn became the most unfavorable, and the maximum deflection was revealed on the glass sheet D. We further analyzed the influence of each factor on the stress and deflection based on the data obtained from the orthogonal test, and the analysis results for the plate center were given in Fig 13.

**Fig 10:**
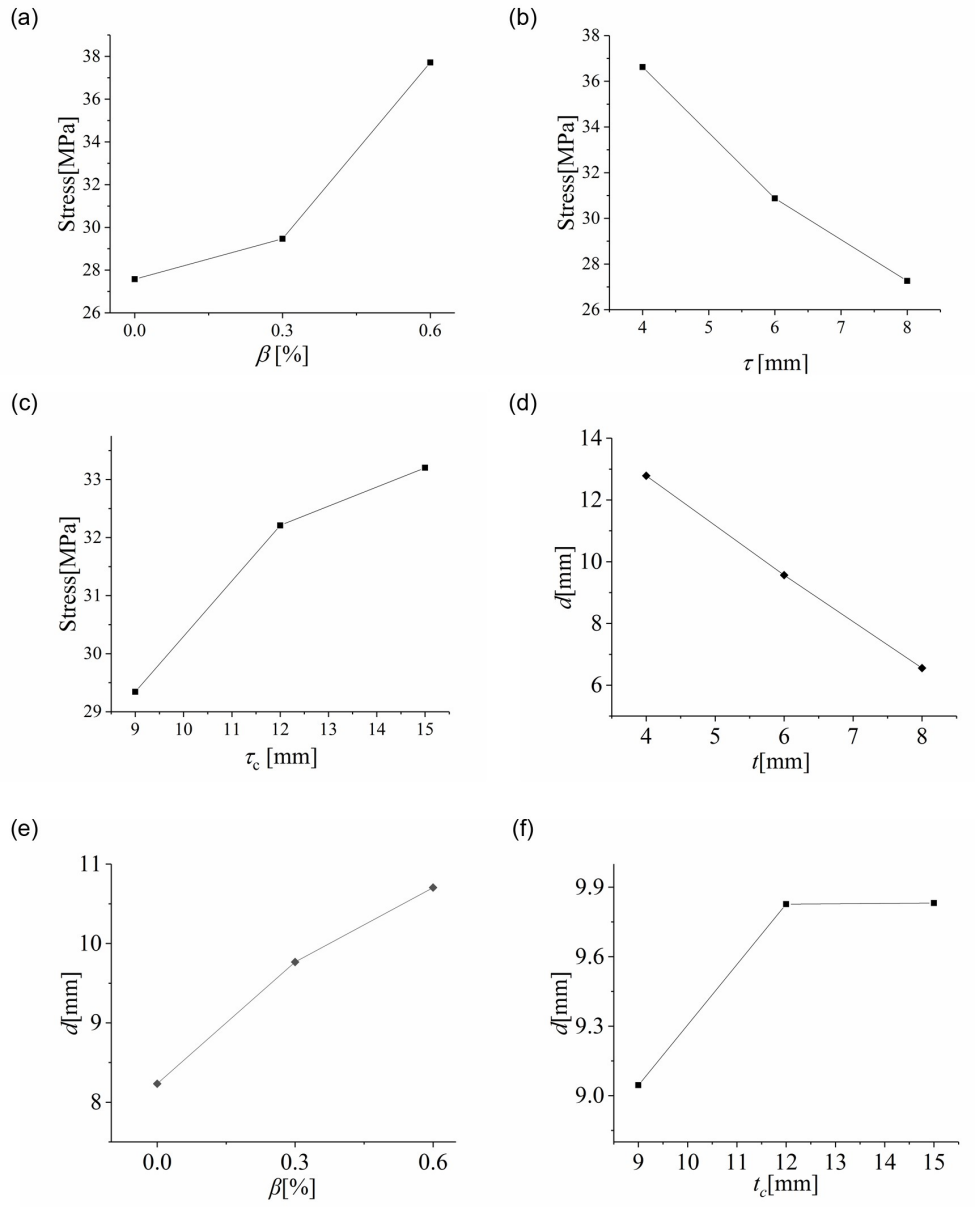
Influence curves of. (a) cold-bent torsion rate, (b) glass thickness, (c) cavity thickness on the plate center stress; (d) cold-bent torsion rate, (e) glass thickness, (f) cavity thickness on the plate center deflection.

The range *R*_*j*_ in the orthogonal experiment analysis is the difference between the maximum and minimum values of the average value of each level under factor *j*, which can show the influence of factor *j*. In this test, the range of cold bending distortion rate, glass thickness and cavity thickness are *R*_*β*_, *R*_*t*_ and *R*_*tc*_, respectively. As we can see from the figures, cold-bent torsion rate could cause the greatest influence on the stress of the plate center with a stress range (*R*_*β*_) of 10.14, the largest among all curves. Besides, it can be seen from Fig 10(a) that as the cold-bent torsion rate rose, the maximum stress at the center of U-2 grew faster and faster.

The deformation pattern for the samples after cold bending was similar to that after uniform loading, meaning that the cold-bent samples were in the most unfavorable condition of bigger deformation. In addition, the stress revealed a more obvious increase at the plate center as the procedures of cold bending and uniform load continued. It can be suggested that with a larger cold-bent torsion rate, the working states of the samples would grow more and more adverse with their internal stress rising higher and higher. Similar relationships can be found on deflection, as shown in Fig 10(a), but it turns out that deflection tends to rise in a linear manner.

Among all the factors studied, glass thickness possessed the greatest impact on the deflection of the plate center with the *R*_*β*_ = 6.23. Both stress and deflection at the plate center tend to exhibit a linear decrease as the plate thickness rose, indicating the weakening effect of glass thickness on stress and deflection under a uniform load, as shown in Fig 10(b) and 10(e). Such phenomenon can be explained by the glass rigidity, which is greatly affected by glass thickness (the rigidity of a monolithic glass plate is proportional the cubic of its thickness). Generally speaking, the more rigid a plate is, the smaller stress and deflections it exhibits.

Judging from Fig 10(c) and 10(f), an increase in cavity thickness would cause the maximum stress and deflection at the plate center to rise. The corresponding range *R*_*tc*_ for stress and deflection were 3.00 and 0.784 respectively, indicating that the effect of cavity thickness was the smallest among all factors studied here. According to the simplified model of spacer bars for insulating glass [20], with the spacer becoming higher, a decrease in decomposed tensile and bending stiffness, which was trivial compared to the overall glass thickness, will appear. Therefore, the impact brought by cavity thickness on stress and deflection was the smallest.

## 4. Finite element simulation

### 4.1. Establishment of numerical model

**Fig 11:**
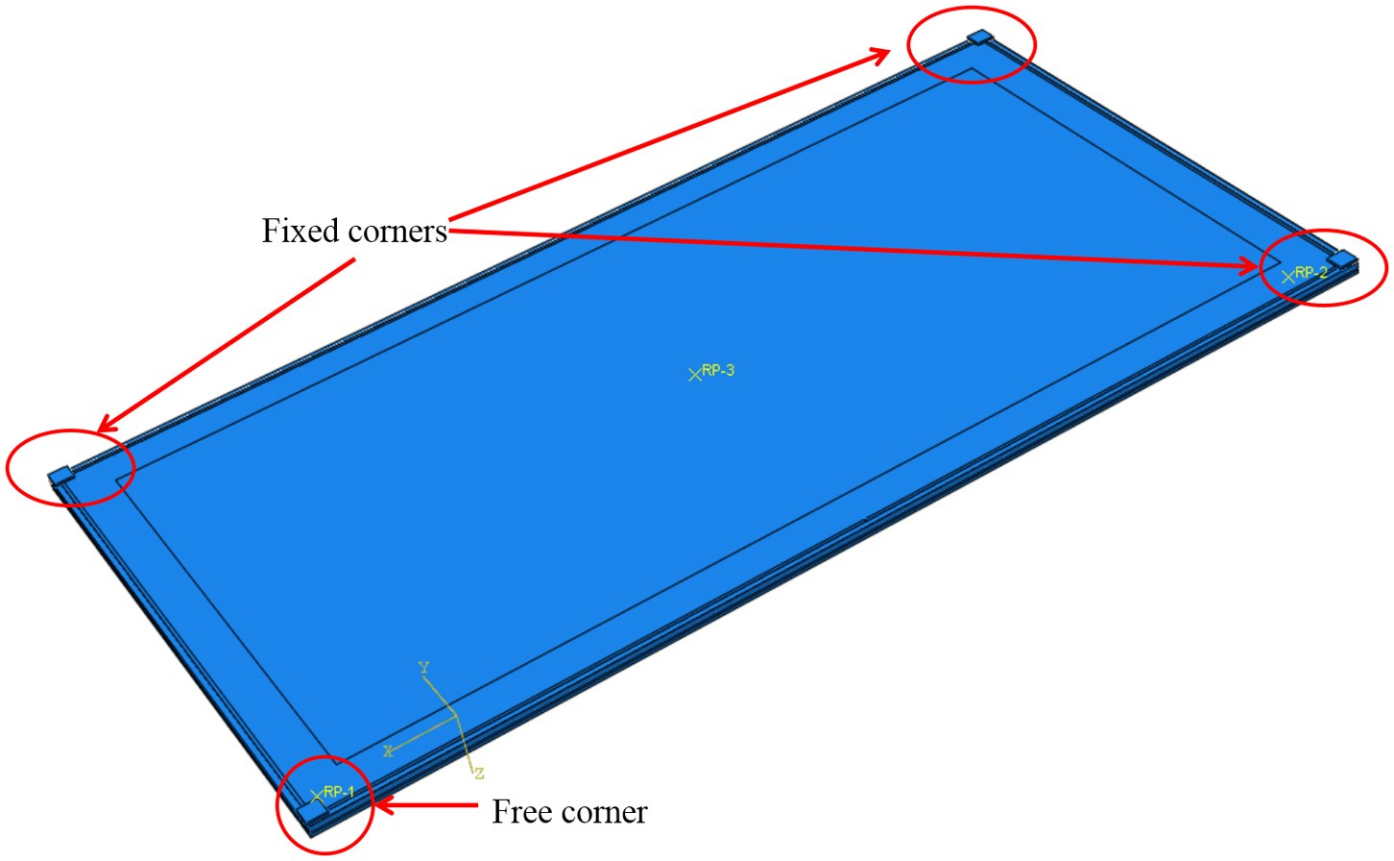
Sample assembly in FEA.

The finite element software ABAQUS was used to simulate and analyze the insulating glass. The components in the numerical model include elastic materials (such as glass, aluminum spacer bars, cold bending loading device) and hyperelastic materials (such as silicone sealants, rubber, etc.). The properties of each material were shown in Table 3. Elastic materials were set in accordance to the Chinese code [18], and the mesh division forms adopted by the components were generated by the medial axis sweeping algorithm. Among all the components, the glass sheets and aluminum spacer bars adopted a cell mesh structured with a reduced integral quadrilateral shell. For hyperelastic materials, no specific elastic modulus value exists, and their properties were related to many factors. In finite element analysis (FEA), a common model to simulate rubber was the Mooney-Rivlin model because it can produce small strain in the test, and the material parameters were set as C_10_ = 0.142, C_01_ = 0.011 in this experiment based on previous study [21]. When rubber produces a large strain, it would harden, and the silicone sealants in the tests here would produce a large strain under external force, excluding the Mooney-Rivlin model as the choice for simulation. Instead, the Yeoh model would be a better choice in FEA for silicone sealants, and the recommended parameters for the material’s mechanical properties were set as C_1_ = 1.077, C_2_ = -0.298, C_3_ = 0.238 that were given by a previous research [22] and integrated in the current study. The C3D8RH mixed solid cell was used for simulation. In addition, butyl tapes had little impact on the mechanics of insulating glass [20, 23]. The final model assembly was shown in Fig 11. For the mesh resolution, the elements of the glass were 20mm in size, the aluminum spacer bars were set to 15mm, the rubber was set to 10mm, and the silicone rubber was set to 10mm.

**Table 3:** The material properties.

Material	Element type	Elastic modulus[N/mm^2^]	Poisson ratio	Density[ton/m^3^]
Glass	Shell	72000	0.20	2.56
Aluminum	Shell	70000	0.33	2.80
Steel	Solid	206000	0.20	7.85

We adopted surface-to-surface contact for the connection between the glass plate and the rubber pad. The aluminum spacer bars, silicone sealants and glass were regarded as a whole by tie constraints. The spacer bars and the two glass pieces were enclosed as a cavity, and ABAQUS was used to simulate constant pressure with its function of “fluid cavity”. The specified cavity pressure was set to be 0.101MPa, and the ideal gas constant value was 8.314J/(mol·K).

### 4.2. Validation of the numerical model

**Fig 12:**
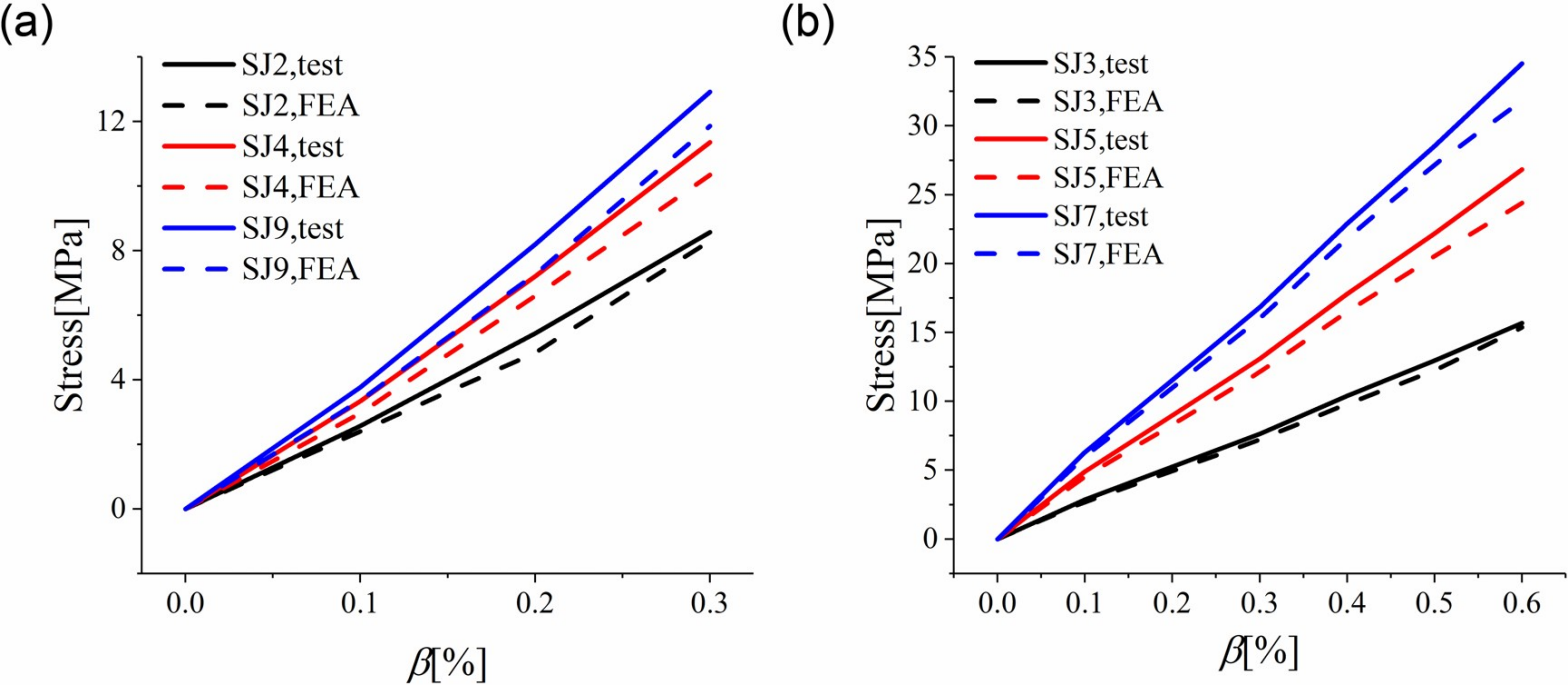
Maximum stress found in experiment and simulation on cold bending process. (a) SJ2, SJ4, SJ9; (b) SJ3, SJ5, SJ7.

Table 4 showed results from numerical simulation and actual tests about the cold-bent control stress, revealing a deviation below 9.05%. The simulated cold-bent control stress was near to the cold-bent corner on the short side of the surface D-2, which was the same as the test. During cold bending loading, the change in control stress with cold bending displacement can be characterized as the relationship between cold-bent torsion rate and stress. The curves of cold-bent torsion rate and control stress in cold bending in numerical simulation and actual tests were shown in Fig 12. It can be seen from the simulation section that as the cold-bent torsion rate rises, the relationship between the control stress and curvature in cold bending was approximately linear. It can be known from the previous analysis that the influence of the thickness of the cavity was trivial, so stress was affected mainly by the glass stiffness and the stress of large glass thickness was greater than that of small thickness.

**Table 4:** Maximum stress on surface D-2 of simulation and test results.

Specimen	*σ*_1,TEST_[MPa]	*σ*_1,FEA_[MPa]	Deviation
SJ2	8.57	8.311	3.02%
SJ3	15.68	15.365	2.01%
SJ4	11.35	10.348	8.83%
SJ5	26.83	24.403	9.05%
SJ7	34.52	31.822	7.82%

**Fig 13:**
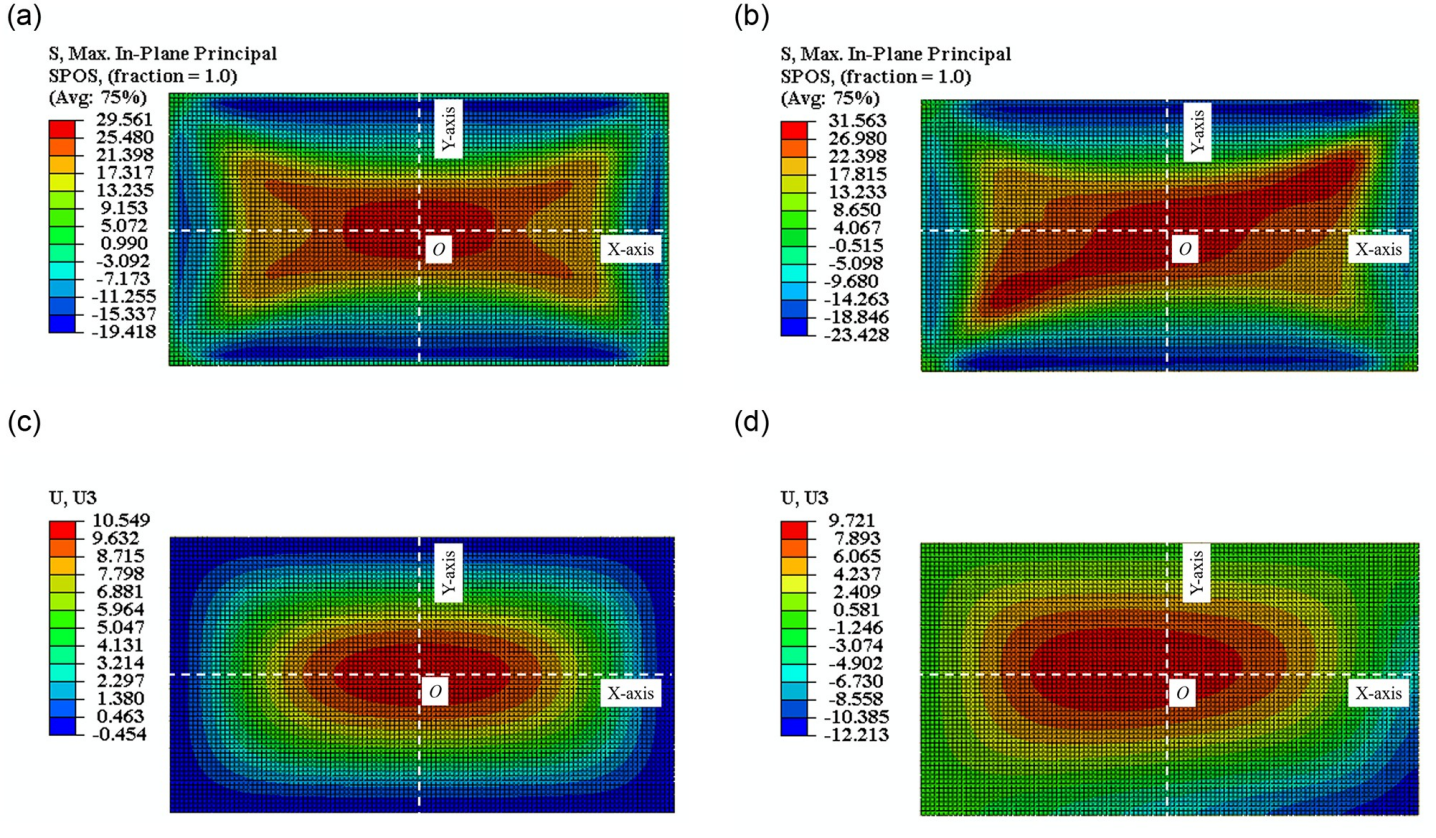
The nephogram in FEA of. (a)SJ1, (b)SJ2 of stress; (c)SJ1, (d)SJ2 of deflection.

The simulation revealed an identical pattern of control stress transfer to the experiments, which was from the cold-bent control point to the plate center in cold-bent glass after applying a uniform load, and the maximum stress was also found on the surface U-2. The simulated principal stress values on U-2 were compared with those from the tests in Table 5, and the deviation was 10.28%, showing a general consistency between the two data sets. In terms of stress distribution, the FEA results for SJ1 (non-cold-bent) and SJ2 (cold-bent) were shown in Fig 13(a) and 13(b).

**Table 5:** Comparison of central stress of the surface U-2 between simulation and test results.

Specimen	*σ*_1,TEST_[MPa]	*σ*_1,FEA_[MPa]	Deviation
SJ1	32.07	29.561	7.82%
SJ2	34.12	31.563	7.49%
SJ3	43.68	40.720	6.78%
SJ4	25.82	23.166	10.28%
SJ5	39.33	37.882	3.68%
SJ6	27.47	26.419	3.83%
SJ7	30.14	28.650	4.94%
SJ8	23.18	21.979	5.18%
SJ9	28.46	27.015	5.08%

Table 6 showed the plate center deflections obtained from FEA and actual tests, and Fig 13(c) and 13(d) showed the deflection nephograms of SJ1 and SJ2. If we took the direction of the uniform load as positive, the maximum deflection on SJ1 was at the plate center and stress at edges was pointing negative, and the maximum deflection on SJ2 was near to the plate center. In addition, the deflection of the cold bending corner was also negative, which was dominantly from the cold-bent displacement before applying load. The deviations between FEA and experiment were 9.83%, so low that the accuracy of FEA in uniform load test was verified.

**Table 6:** Comparison of plate central deflection between simulation and test.

Specimen	*σ*_1,TEST_[MPa]	*σ*_1,FE_[MPa]	Deviation
SJ1	10.816	10.549	2.47%
SJ2	10.599	9.721	8.28%
SJ3	9.426	9.000	4.52%
SJ4	6.787	6.120	9.83%
SJ5	5.612	5.100	9.12%
SJ6	8.160	7.733	5.23%
SJ7	2.017	1.928	4.41%
SJ8	5.723	5.656	1.17%
SJ9	4.404	4.095	7.02%

## 5. Conclusions

In this report, we performed experimental studies on the mechanical response of anticlastic cold bending insulating glass under different glass thicknesses, cold-bent torsion rates and cavity thicknesses. Besides, we conducted numerical simulations for the tests, and verified the accuracy of such simulations by comparing their results with those from the experiments. What we found after the experiments and simulations could be concluded as the following:

1. After anticlastic cold bending, two glass sheets would exhibit an uneven stress distribution. The cold-bent control stress is on the surface with direct loads from cold bending, and it is close to the cold-bent corner on the short edge.
2. On the glass plate, the control stress is transferred from the parts around the corner to the center when the uniform load plays a leading role in generating stress. This transfer could occur under a relatively small load with a small cold-bent torsion rate, and the maximum stress is controlled by cold bending as the cold-bent torsion rate becomes larger with a thicker glass plate.
3. For the cold-bent insulating glass coupled with uniform load, the cold-bent torsion rate is the most influential on aggravating the plate center stress. The insulating glass would get more and more unfavorable due to a growing deformation in the same direction resulted from cold bending and uniform load. With large glass thickness, glass stress and deflection could be significantly reduced, but effects brought by changes in cavity thickness are rather trivial.
4. Numerical simulation is verified to be an effective approach to study the mechanical response of cold bending glass.

## Supporting information

S1 Table Cold-bent maximum principal stress of glass U and D. (XLSX)

S1 File CAD draw file of nomination of insulating glass surfaces. (DWG)

S2 File PDF file of nomination of insulating glass surfaces. (PDF)

S3 File 3D-CAD draw of the test device. (DWG)

S4 File CAD draw file of the layout of measuring nodes. (DWG)

S5 File PDF files of the layout of measuring nodes. (PDF)

S6 File MATLAB coding file of Fig 6a. (M)

S7 File MATLAB coding file of Fig 6b. (M)

S8 File MATLAB coding file of Fig 6c. (M)

S9 File MATLAB coding file of Fig 6d. (M)

S10 File MATLAB coding file of Fig 6e. (M)

S11 File MATLAB coding file of Fig 6f. (M)

S12 File MATLAB coding file of Fig 7a. (M)

S13 File MATLAB coding file of Fig 7b. (M)

S14 File MATLAB coding file of Fig 7c. (M)

S15 File MATLAB coding file of Fig 7d. (M)

S16 File MATLAB coding file of Fig 7e. (M)

S17 File MATLAB coding file of Fig 7f. (M)

S18 File Origin source file of Fig 8a. (OPJ)

S19 File Origin source file of Fig 8b. (OPJ)

S20 File Origin source file of Fig 8c. (OPJ)

S21 File Origin source file of Fig 8d. (OPJ)

S22 File Origin source file of Fig 8e. (OPJ)

S23 File Origin source file of Fig 8f. (OPJ)

S24 File Origin source file of Fig 9a. (OPJ)

S25 File Origin source file of Fig 9b. (OPJ)

S26 File Origin source file of Fig 9c. (OPJ)

S27 File Origin source file of Fig 9d. (OPJ)

S28 File Origin source file of Fig 9e. (OPJ)

S29 File Origin source file of Fig 9f. (OPJ)

S30 File Origin source file of Fig 10a. (OPJ)

S31 File Origin source file of Fig 10b. (OPJ)

S32 File Origin source file of Fig 10c. (OPJ)

S33 File Origin source file of Fig 10d. (OPJ)

S34 File Origin source file of Fig 10e. (OPJ)

S35 File Origin source file of Fig 10f. (OPJ)

S36 File Origin source file for Fig 12a. (OPJ)

S37 File Origin source file for Fig 12b. (OPJ)

S38 File CAD draw file of loading history curves. (DWG)

S39 File CAD draw file of Fig 2a. (DWG)

S40 File CAD draw file of Fig 2b. (DWG)

S41 File CAD draw file of Fig 2c. (DWG)
