# Supplementary figures and images for: Study on the mechanical response of anticlastic cold bending insulating glass and its coupling effect with uniform load
Source: PLoS One. 2021 Apr 23;16(4):e0250463. doi: 10.1371/journal.pone.0250463 (PMC8064554; doi:10.1371/journal.pone.0250463)

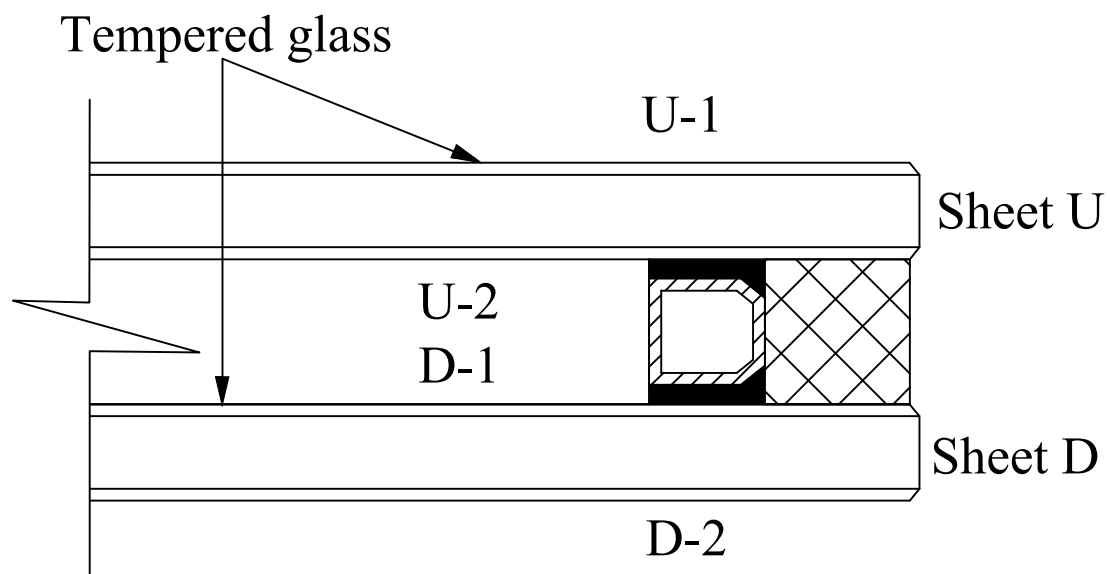

- 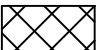 Silicone sealant
- 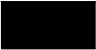 Butyl tape
- 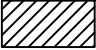 Aluminum spacer bar

Supplement: S2 File — (PDF) [file pone.0250463.s003.pdf]

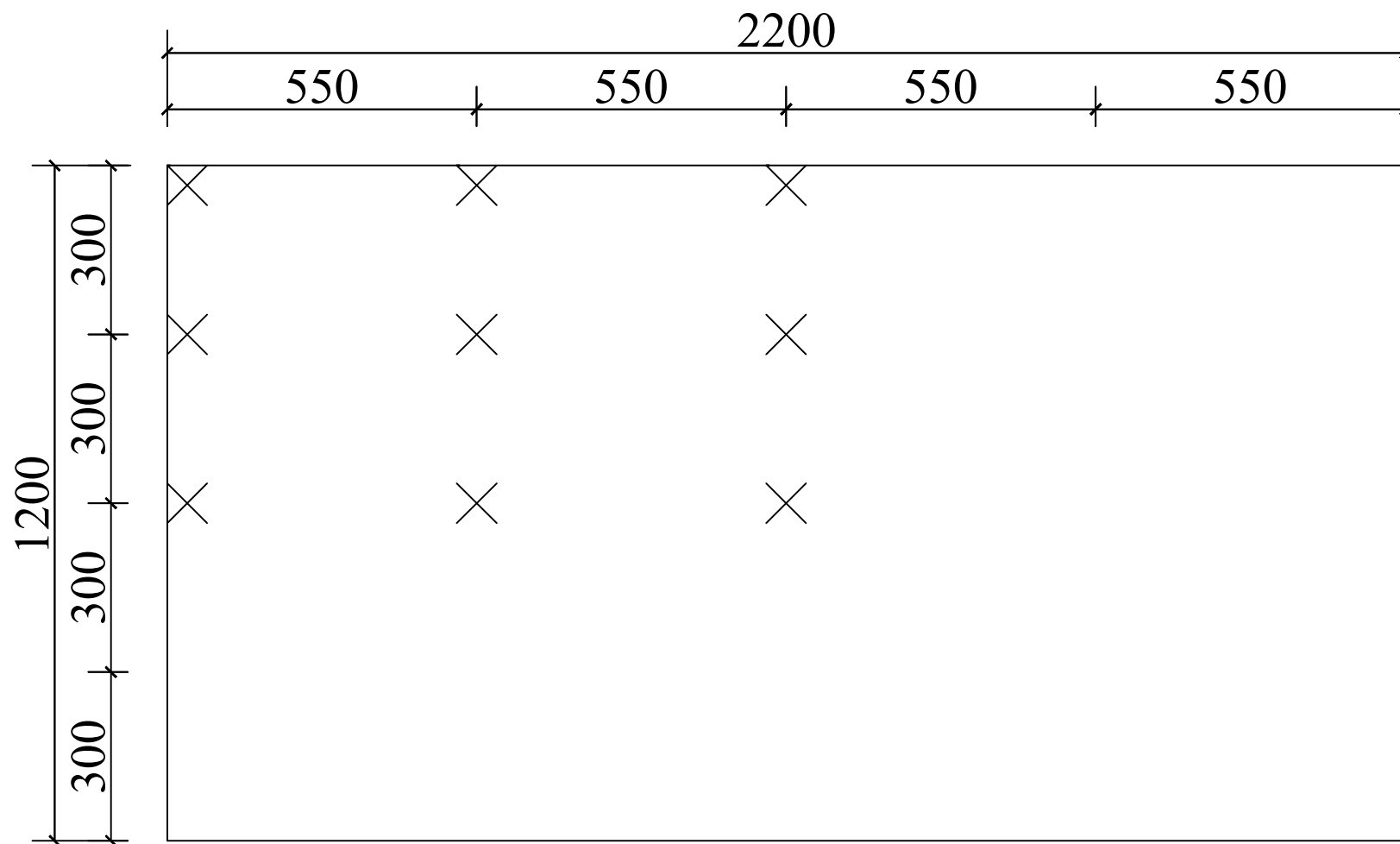

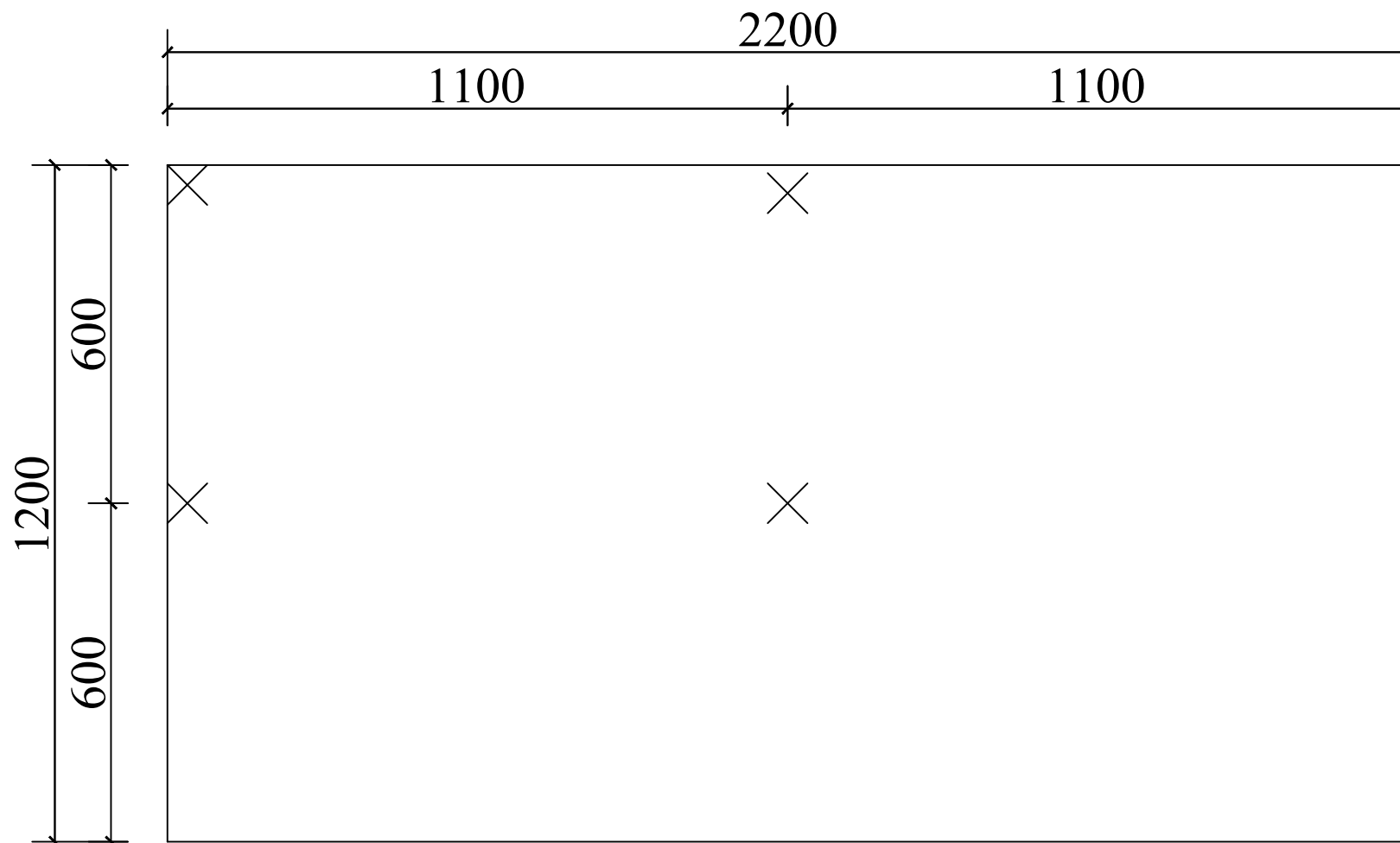

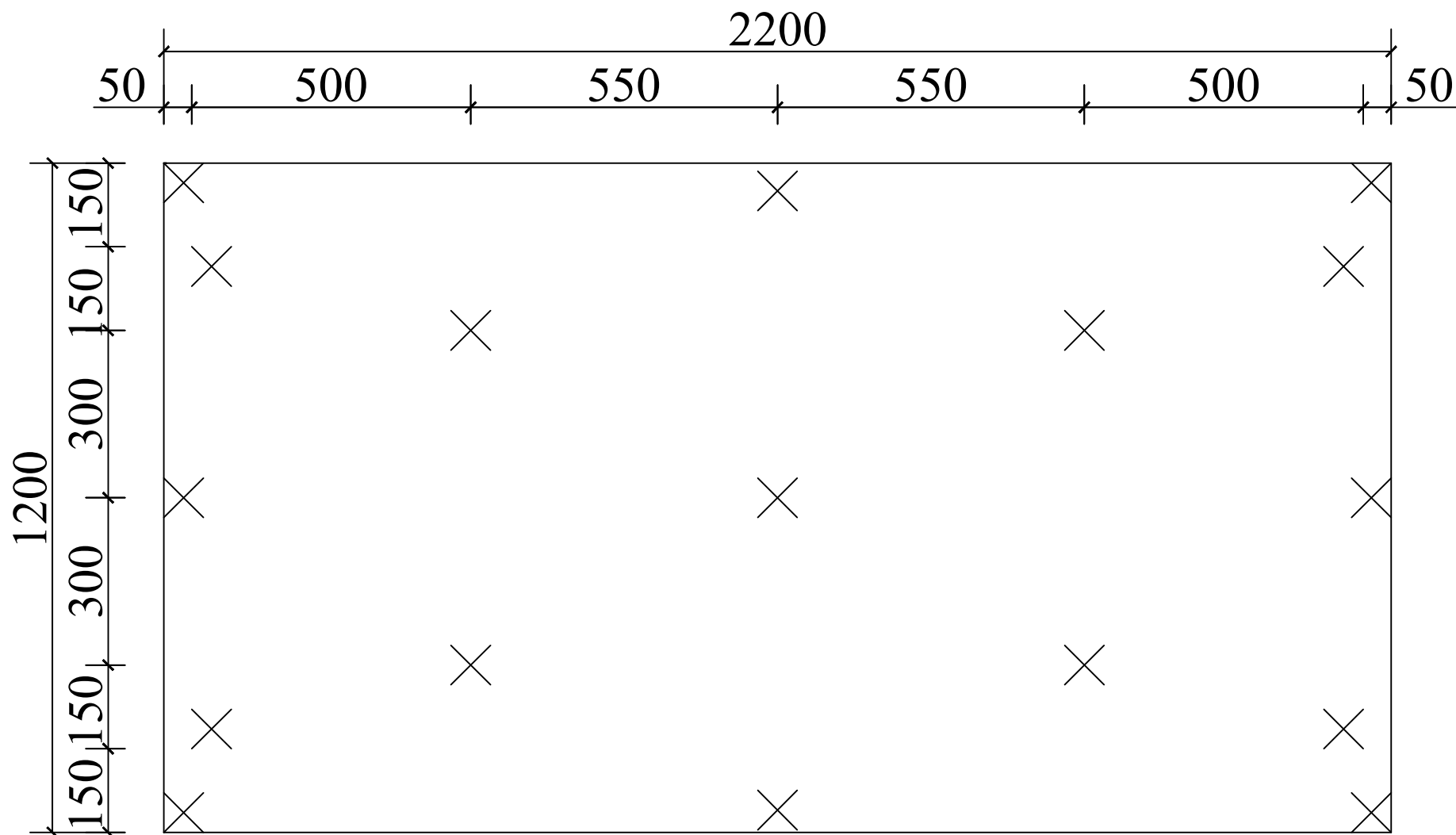

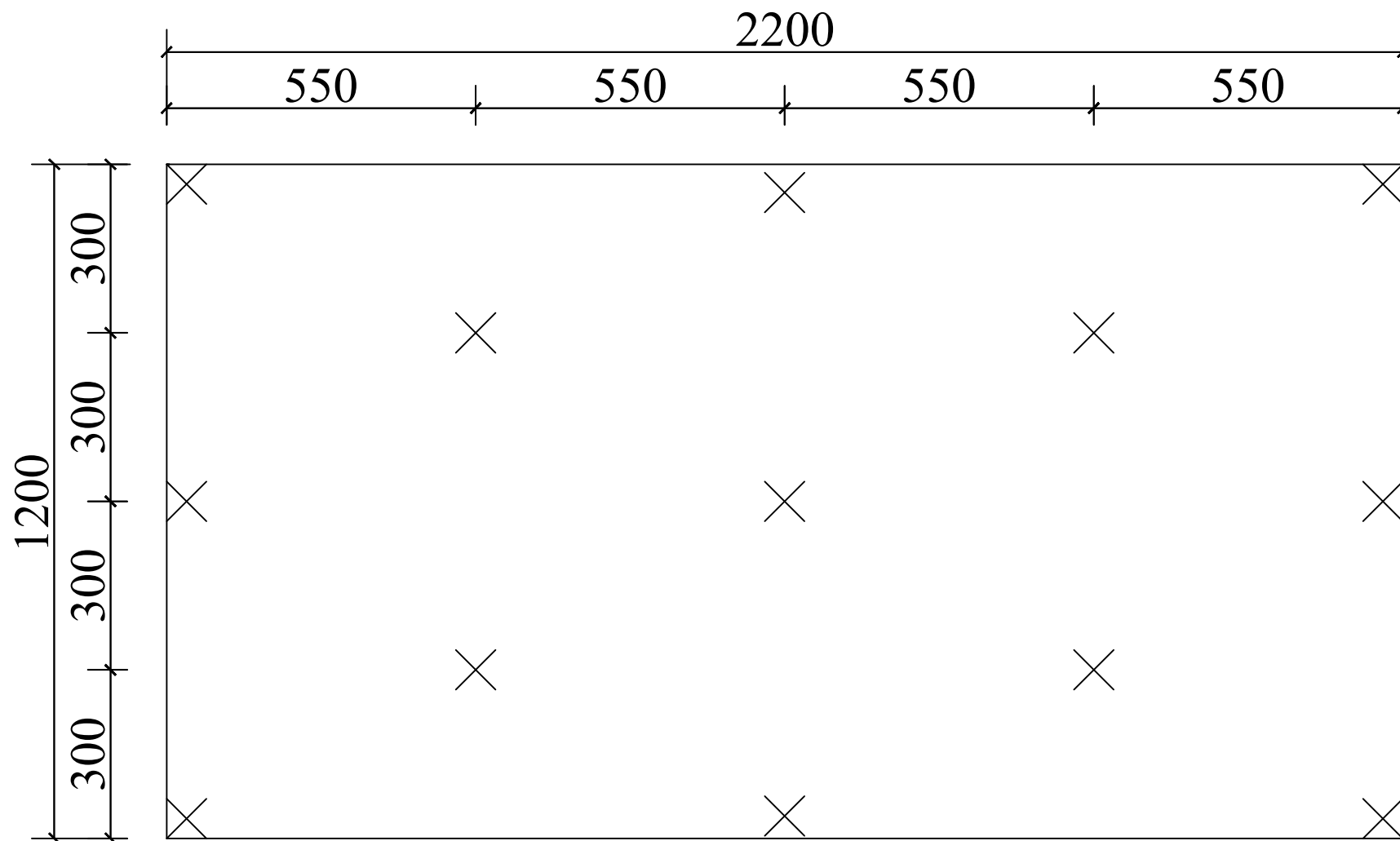

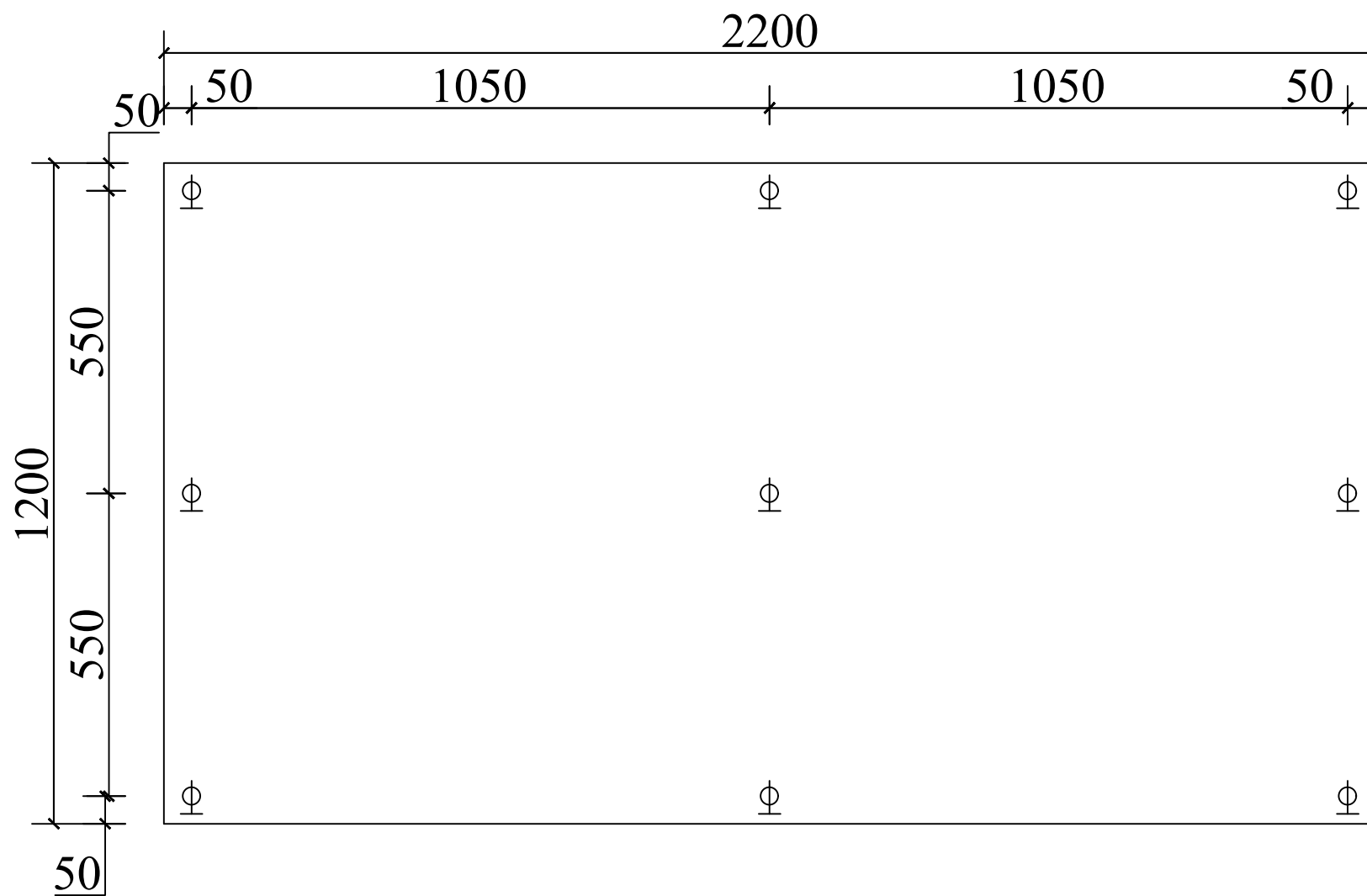

Supplement: S5 File — (PDF) [file pone.0250463.s006.pdf]
